# The association between the triglyceride–glucose index and the risk of cardiovascular disease in US population aged ≤ 65 years with prediabetes or diabetes: a population-based study

**DOI:** 10.1186/s12933-024-02261-8

**Published:** 2024-05-13

**Authors:** Chang Liu, Dan Liang

**Affiliations:** 1 School of Medicine, Nankai University, Tianjin, China; 2 Department of Endocrine, People’s Hospital of Chongqing Liang Jiang New Area, Chongqing, China; 3 The West China College of Medicine, Sichuan University, Chengdu, China

**Keywords:** TyG index, CVD, Diabetes, Pre-diabetes

## Abstract

**Background:**

The relationship between the triglyceride–glucose (TyG) index and the risk of cardiovascular disease (CVD) in the U.S. population under 65 years of age with diabetes or prediabetes is unknown. The purpose of this study was to investigate the relationship between baseline TyG index and CVD risk in U.S. patients under 65 years of age with diabetes or prediabetes.

**Methods:**

We used data from the 2003–2018 National Health and Nutrition Examination Survey (NHANES). Multivariate regression analysis models were constructed to explore the relationship between baseline TyG index and CVD risk. Nonlinear correlations were explored using restricted cubic splines. Subgroup analysis and interaction tests were also conducted.

**Results:**

The study enrolled a total of 4340 participants with diabetes or pre-diabetes, with a mean TyG index of 9.02 ± 0.02. The overall average prevalence of CVD was 10.38%. Participants in the higher TyG quartiles showed high rates of CVD (Quartile 1: 7.35%; Quartile 2: 10.04%; Quartile 3: 10.71%; Quartile 4: 13.65%). For CVD, a possible association between the TyG index and the risk of CVD was observed. Our findings suggested a linear association between the TyG index and the risk of CVD. The results revealed a U-shaped relationship between the TyG index and both the risk of CVD (P nonlinear = 0.02583) and CHF (P nonlinear = 0.0208) in individuals with diabetes. Subgroup analysis and the interaction term indicated that there was no significant difference among different stratifications. Our study also revealed a positive association between the TyG index and comorbid MetS in the U.S. population under 65 years of age with prediabetes or diabetes.

**Conclusions:**

A higher TyG index was linked to an increased likelihood of CVD in the U.S. population aged ≤ 65 years with prediabetes and diabetes. Besides, TyG index assessment will contribute to more convenient and effective screening of high-risk individuals in patients with MetS. Future studies should explore whether interventions targeting the TyG index may improve clinical outcomes in these patients.

**Supplementary Information:**

The online version contains supplementary material available at 10.1186/s12933-024-02261-8.

## Introduction

Cardiovascular disease (CVD) continues to increase in morbidity and mortality This upward trend imposes a substantial burden on both the healthcare system and overall human well-being, thereby emerging as a major public health issue of global concern [1, 2]. Findings from a comprehensive Global Burden of Disease study encompassing 204 countries and territories spanning the years 1990 to 2019 revealed a noteworthy surge in the prevalence of CVD [3]. The population afflicted by CVD has more than doubled, escalating from 271 million in 1990 to 573 million in 2019. Concurrently, the fatalities attributable to CVD have surged from 12.1 million in 1990 to 18.6 million. Furthermore, global patterns in disability-adjusted life years (DALYs) and years of life lost exhibit a significant and concerning uptick during this period [3]. Hypertension, unhealthy lifestyle, dyslipidemia, and diabetes are common risk factors for CVD [4]. Notably, CVD prevalence and mortality are higher in low- and middle-income countries compared to high-income countries [5]. Hence, the proactive identification and screening of individuals at an early stage of cardiovascular risk, coupled with the timely implementation of interventions to address risk factors, stand as pivotal measures to curtail the incidence of cardiovascular events and mitigate the looming threat to human life.

Insulin resistance (IR) is a pathophysiological disorder characterized by defective insulin regulation of glucose metabolism in tissue cells, which is primarily denoted by a diminished sensitivity and responsiveness of the body to insulin, potentially resulting in metabolic irregularities such as hyperglycemia, hyperlipidemia, and obesity [6–8]. Notably, it emerges as a novel and independent risk factor for CVD. TyG index serves as a marker utilized for evaluating insulin resistance (IR) based on fasting triglyceride and glucose levels [9]. In comparison to the hyperinsulinemic–euglycemic clamp technique, the TyG index stands out for its cost-effectiveness and ready availability [10, 11]. It has been demonstrated that the TyG index outperforms the Homeostasis Model of Insulin Resistance (HOMA-IR) in assessing IR [12]. Moreover, associations have been observed between the TyG index and adverse clinical outcomes in individuals with CVD [13, 14], heart failure [8], ischemic stroke [15], atherosclerosis [16, 17], and non-alcoholic fatty liver disease (NAFLD) [18].

The TyG index is a useful, low-cost predictive marker of the risk of cardiovascular and coronary heart disease in the non-diabetic population [19]. Additionally, as an IR marker, the TyG index exhibits a close association with type 2 diabetes, with a prevalence of cardiovascular disease as high as 32.2% in individuals with type 2 diabetes [20]. However, the relevance of the TyG index to the risk of early cardiovascular disease (CVD) in patients with diabetes or prediabetes remains contentious, and higher TyG indices have been reported in younger patients [21]. Regrettably, few large-scale studies have explored the early CVD risk in young patients with diabetes or prediabetes. Our study endeavors to ascertain whether the TyG index holds prognostic value for identifying early cardiovascular risk in diabetic or prediabetic patients below the age of 65.

## Materials and methods

### Study design

The National Health and Nutrition Examination Survey (NHANES), an ongoing project employing a complex, multistage probability sampling design to evaluate the health and nutritional status of the ambulatory population in the U.S., received approval from the Institutional Review Board of the National Center for Health Statistics (NCHS), with informed consent obtained from all participants. NHANES gathers questionnaire data through interviews, performs health screenings at mobile examination centers (MECs), and collects samples for laboratory testing. A comprehensive overview of the NHANES study and its data is accessible online at https://www.cdc.gov/nchs/nhanes/.

### Study population

**Fig. 1:**
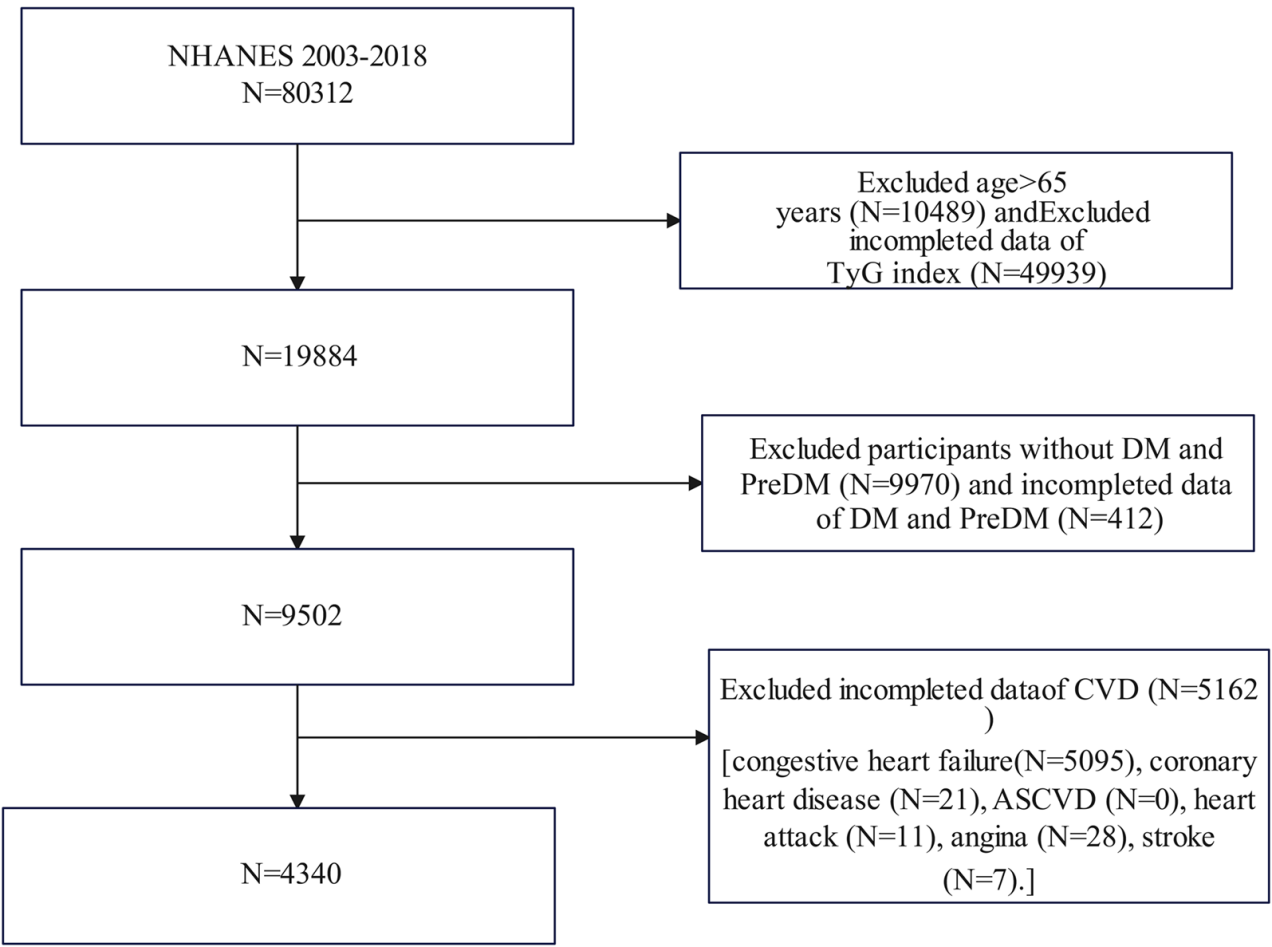
Flowchart of the sample selection from National Health and Nutrition Examination Survey (NHANES) 2003–2018

We utilized NHANES survey cycles from 2003 to 2018, as these surveys provided comprehensive data on the TyG index and various cardiovascular conditions, including congestive heart failure (CHF), coronary heart disease (CHD), atherosclerotic cardiovascular disease (ASCVD), heart attack, angina, and stroke. Initially, 80,312 participants were enrolled in the study. After excluding individuals aged > 65 years (N = 10,489), those without diabetes or pre-diabetes (N = 9970), and those with missing data on the TyG index (N = 49,939), diabetes or pre-diabetes (N = 412) and specific cardiovascular conditions (N [CVD] = 5162 [CHF: 5095; CHD: 21; ASCVD: 0; heart attack: 11; angina: 28; stroke: 7]), our final analysis included 4340 eligible participants (Fig. 1).

### Assessment of triglyceride–glucose index

TyG was utilized as an exposure variable, and we computed the TyG index using the formula: Ln [triglycerides (mg/dL) * fasting glucose (mg/dL)/2]. Both triglyceride and fasting glucose concentrations were determined through an enzymatic assay employing an automatic biochemistry analyzer. Serum triglyceride concentration was measured using the Roche Modular P and Roche Cobas 6000 chemistry analyzers while fasting plasma glucose was assessed through the hexokinase-mediated reaction using the Roche/Hitachi Cobas C 501 chemistry analyzer.

### Assessment of cardiovascular disease

The medical conditions section, identified by the variable name prefix MCQ, encompasses self- and proxy-reported personal interview data that spans a diverse array of health conditions and medical history for both children and adults. This section incorporates questions such as'Has a doctor or other health professional ever told you/SP that you/him/her… had CHF, CHD, angina (also called angina pectoris), heart attack (also called myocardial infarction), stroke, etc.?' These questions were denoted as MCQ160B-F in the household questionnaires administered during home interviews. Participants responding'yes' to any of these questions were classified as having a history of CVD. We established a composite endpoint for CVD, encompassing CHD, ASCVD, angina, stroke, and CHF as primary outcomes. Additionally, events related to CHD, ASCVD, angina, stroke, and CHF were separately analyzed as secondary outcomes.

### Assessment of diabetes and prediabetes

Diabetes was defined as either treatment or medical diagnosis of hyperglycemia with hemoglobin A1c ≥ 6.5%, fasting plasma glucose (FPG) ≥ 126 mg/dL, or a 2-h blood glucose ≥ 200 mg/dL [22]. Prediabetes is identified by self-reported prediabetes status or having FPG between 100 and 125 mg/dL, or HbA1c between 5.7 and 6.4% [23].

### Assessment of metabolic syndrome (MetS)

MetS was defined according to the National Cholesterol Education Program/Adult Treatment Panel III criteria (NCEP-ATP III) [24, 25]. Individuals with three or more of the following conditions were classified as having MetS: (1) Central obesity: Waist circumference (WC) exceeding 102 cm in men or 88 cm in women. (2) Elevated triglyceride (TG) levels: Equal to or greater than 1.7 mmol/L (150 mg/dL). (3) Reduced high-density lipoprotein cholesterol (HDL-C) levels: Less than 1.03 mmol/L (40 mg/dL) in men or less than 1.29 mmol/L (50 mg/dL) in women. (4) Elevated blood pressure: Systolic blood pressure (SBP) equal to or greater than 130 mmHg, or diastolic blood pressure (DBP) equal to or greater than 85 mmHg. (5) Impaired fasting glucose: FPG equal to or greater than 100 mg/dL.

### Section of covariates

Data on various demographic and health-related factors were collected through NHANES household interviews. This encompassed details such as age, gender, race/ethnicity, educational level, family income, smoking status, alcohol consumption, and disease status. Body Mass Index (BMI) was computed by dividing weight (in kilograms) by the square of height (in meters). Participants were classified as normal weight (< 25 kg/m^2^), overweight (25–29.9 kg/m^2^), or obese (≥ 30 kg/m^2^) based on their BMI. Blood pressure (BP) measurements were obtained by physicians using mercury sphygmomanometers following a standard protocol in the Mobile Examination Center (MEC). Participants presenting any of the following conditions on both arms were excluded from the examination: rash, gauze dressing, plaster, edema, paralysis, tubal, open ulcer or wound, arm blight, arteriovenous shunt, and mastectomy. BP measurements were performed in the right arm unless a specific condition precluded its use or the participant reported any reason why BP measurements should not be performed in the right arm. Each participant underwent 1–4 BP readings in the study, and individuals without any BP readings were excluded. For those with only one BP reading, it served as the final record. When multiple BP readings were available, the first reading was always excluded, and the BP record represents the average of the subsequent readings. Hypertension was defined as the use of antihypertensive medications, a medical diagnosis of hypertension, or three consecutive measurements of systolic blood pressure ≥ 140 mmHg or diastolic blood pressure ≥ 90 mmHg [26]. Clinical indicators, including serum creatinine, serum uric acid, fasting glucose, HbA1c, TG, total cholesterol (TC), low-density lipoprotein cholesterol (LDL-C), and HDL-C, urinary albumin: creatinine ratio (ACR), and estimated-glomerular filtration rate (eGFR) were assessed in the NHANES laboratory.

### Statistical analysis

According to NHANES analytic guidelines, statistical analyses were performed with appropriate sampling weights and accounting for complex multistage cluster surveys. Continuous variables were presented as means ± standard deviations, while categorical variables were expressed as percentages. Participants, categorized based on the TyG index quartiles, were compared utilizing a weighted Student’s t-test for continuous variables or a weighted chi-square test for categorical variables. Multivariate logistic regression was utilized to investigate the association between the TyG index (independent variable) and the risk of CVD (dependent variable) through three distinct models for statistical inference. In model 1, no covariates were adjusted. Model 2 was adjusted for gender, age, and race. Model 3 involved adjustments for age, gender, race, education level, family income-poverty ratio (PIR), BMI, serum creatinine, serum uric acid, TC, LDL-C, HDL-C, ACR, eGFR, systolic blood pressure, diastolic blood pressure, hypertension, smoking status, and alcohol consumption. In sensitivity analyses, we categorized the TyG index into quartiles to assess the robustness of the results and examined the risk of CVD across these quartiles. Additionally, we employed restricted cubic spline (RCS) analysis with three piecewise points to explore potential nonlinear relationships between the TyG index and the CVD risk. For subgroup analysis concerning the association between the TyG index and the likelihood of CVD, we stratified the data by gender (male/female), BMI (normal weight/overweight/obesity), hypertension (yes/no), alcohol use (yes/no) and smoking status (never/former/now). These stratified factors were also considered as potential effect modifiers. In addition, we also used multivariate logistic regression to investigate the association between the TyG index and the likelihood of MetS in individuals under 65 years of age with prediabetes or diabetes. No were adjusted in Model 1. Model 2 was adjusted for gender, age, and race. Model 3 was adjusted for age, gender, race, education level, PIR, BMI, serum creatinine, serum uric acid, ACR, eGFR, smoking, and alcohol consumption status. For subgroup analysis concerning the association between the TyG index and MetS in individuals under 65 years of age with prediabetes or diabetes, we stratified the data by gender (male/female), BMI (normal weight/overweight/obesity), alcohol use (yes/no) and smoking status (never/former/now). A significance level of two-sided *P* < 0.05 was utilized to indicate statistical significance. All analyses were performed using R version 4.3.2 (http://www.R-project.org, The R Foundation).

## Results

### Baseline characteristics of study participants

In this study, 4340 participants were enrolled, with an average age of 48.48 ± 0.24 years. Among them, 54.96% were male, and 45.04% were female. The mean TyG index was 9.02 ± 0.02. The overall prevalence of CVD was 10.38% and this prevalence decreased as the TyG index increased across quartiles (Quartile 1: 7.35%; Quartile 2: 10.04%; Quartile 3: 10.71%; Quartile 4: 13.65%). Participants in higher TyG quartiles exhibited elevated rates of stroke (Quartile 1: 2.18%; Quartile 2: 2.24%; Quartile 3: 2.52%; Quartile 4: 4.69%), CHF (Quartile 1: 1.40%; Quartile 2: 2.96%; Quartile 3: 3.06%; Quartile 4: 4.04%), CHD (Quartile 1: 2.82%; Quartile 2: 4.22%; Quartile 3: 4.41%; Quartile 4: 5.46%), and ASCVD (Quartile 1: 6.87%; Quartile 2: 9.04%; Quartile 3:9.96%; Quartile 4: 12.08%).

Various factors, including age, gender, race, education level, BMI, serum uric acid, TC, LDL-C, HDL-C, ACR, eGFR, systolic blood pressure, diastolic blood pressure, hypertension, smoking status, and alcohol consumption, exhibited significant differences among the TyG index quartiles (all *p* < 0.05). Compared to the lowest TyG index group, participants in the higher TyG index group were significantly more likely to have diabetes and hypertension, elevated serum uric acid, TC, LDL-C, fasting glucose, triglyceride, HbA1c%, BMI, ACR, systolic blood pressure, diastolic blood pressure, and decreased eGFR and HDL-C, more likely to be male, Mexican American, poor education level, and former smokers. No statistically significant differences were observed between quartiles in serum creatinine, PIR, and the risk of heart attack and angina (all *p* > 0.05) (Table 1).

**Table 1:** Weighted baseline characteristics of the study population

TyG index	All participants	Quartile 1 (5.65–8.56)	Quartile 2 (8.56–8.97)	Quartile 3 (8.97–9.44)	Quartile 4 (9.44–12.84)	*p* Value
Age (year)	48.48 (0.24)	46.79 (0.43)	48.31 (0.54)	49.11 (0.47)	49.71 (0.47)	** < 0.0001**
Serum creatinine (mg/dL)	0.88 (0.01)	0.89 (0.02)	0.88 (0.02)	0.87 (0.01)	1.02 (0.02)	0.62
Serum uric acid (umol/L)	344.82 (1.89)	321.29 (3.25)	349.25 (3.17)	352.57 (3.71)	356.94 (3.94)	** < 0.0001**
Total cholesterol (mg/dL)	197.56 (1.09)	182.35 (1.62)	191.70 (1.67)	200.38 (1.66)	216.77 (2.04)	** < 0.0001**
HDL-C (mg/dL)	48.95 (0.35)	58.57 (0.61)	50.56 (0.51)	45.43 (0.43)	40.88 (0.45)	** < 0.0001**
LDL-C (mg/dL)	117.23 (0.87)	109.65 (1.40)	118.35 (1.46)	121.96 (1.55)	119.31 (1.96)	** < 0.0001**
Triglyceride (mg/dL)	164.91 (3.57)	70.64 (0.57)	113.82 (0.99)	164.93 (1.44)	318.45 (9.16)	** < 0.0001**
Fast glucose (mg/dL)	130.21 (0.83)	109.18 (0.73)	117.96 (1.06)	124.28 (1.11)	171.84 (2.83)	** < 0.0001**
HbA1c (%)	6,26 (0.03)	5.72 (0.03)	5.93 (0.05)	6.14 (0.04)	7.31 (0.08)	** < 0.0001**
BMI (Kg/m^2^)	32.39 (0.19)	29.96 (0.33)	32.62 (0.31)	33.31 (0.32)	33.74 (0.29)	** < 0.0001**
ACR (mg/g)	70.32 (7.68)	39.40 (10.57)	40.73 (9.40)	51.99 (11.26)	153.75 (24.96)	** < 0.001**
eGFR (mL/min/1.73 m^2^)	95.88 (0.44)	97.34 (0.72)	96.23 (0.84)	95.75 (0.75)	94.25 (0.71)	**0.03**
Systolic blood pressure (mmHg)	124.92 (0.33)	122.76 (0.67)	123.48 (0.70)	125.73 (0.73)	127.87 (0.64)	** < 0.0001**
Diastolic blood pressure (mmHg)	73.53 (0.26)	72.01 (0.47)	73.40 (0.54)	73.62 (0.48)	75.21 (0.48)	** < 0.0001**
TyG index	9.02 (0.02)	8.20 (0.01)	8.77 (0.00)	9.18 (0.01)	9.99 (0.02)	** < 0.0001**
Gender, % (SE)						** < 0.001**
Female	45.04 (1.86)	50.46 (2.00)	48.13 (2.03)	41.94 (1.91)	39.05 (1.83)	
Male	54.96 (1.86)	49.54 (2.00)	51.87 (2.03)	58.06 (1.91)	60.95 (1.83)	
Races, % (SE)						** < 0.0001**
Mexican American	11.32 (1.12)	8.91 (1.10)	11.08 (1.20)	11.35 (1.08)	14.10 (1.51)	
Non-hispanic black	11.23 (1.08)	16.70 (1.34)	12.23 (1.10)	7.80 (0.80)	8.11 (0.94)	
Non-hispanic white	62.56 (1.82)	59.12 (2.22)	62.69 (2.17)	66.34 (1.79)	61.98 (2.58)	
Others	14.89 (1.34)	15.27 (1.29)	14.00 (1.22)	14.50 (1.29)	15.81 (1.67)	
Educational levels, % (SE)						**0.01**
Less than 9th grade	7.52 (0.87)	5.08 (0.75)	7.50 (0.83)	7.55 (0.84)	10.14 (1.13)	
9–11th grade	12.12 (1.15)	9.69 (0.87)	11.83 (1.24)	13.05 (1.44)	14.05 (1.42)	
High school graduate	25.35 (1.92)	26.35 (1.97)	24.21 (1.97)	26.13 (2.07)	24.73 (1.88)	
Some college or AA degree	31.49 (1.91)	31.32 (2.22)	33.66 (1.93)	28.78 (1.80)	32.11 (1.96)	
College graduate or above	23.52 (2.17)	27.56 (2.38)	22.80 (1.92)	24.50 (1.97)	18.97 (1.92)	
PIR, % (SE)						0.53
< 1	14.26 (1.44)	15.18 (1.35)	16.94 (1.72)	12.58 (1.27)	16.90 (1.57)	
1–4	45.75 (2.21)	48.74 (2.30)	48.63 (2.41)	50.54 (2.49)	48.71 (2.45)	
> 4	33.01 (2.38)	36.08 (2.22)	34.43 (2.65)	36.88 (2.48)	34.39 (2.76)	
BMI, % (SE)						** < 0.0001**
Normal weight	14.09 (1.82)	25.58 (1.88)	13.46 (1.24)	10.13 (1.24)	7.46 (1.01)	
Overweight	28.61 (1.91)	33.11 (1.86)	30.62 (2.05)	25.67 (1.76)	26.48 (1.72)	
Obesity	56.01 (2.10)	41.31 (2.04)	55.93 (2.29)	64.20 (2.05)	66.06 (2.00)	
Smoke, % (SE)						**0.003**
Never	50.62 (2.12)	56.94 (2.23)	50.46 (2.42)	49.44 (2.23)	45.36 (1.83)	
Former	26.96 (1.88)	21.64 (1.85)	27.38 (1.97)	29.36 (2.08)	29.75 (1.61)	
Now	22.38 (1.54)	21.42 (1.69)	22.16 (1.63)	21.19 (1.52)	24.89 (1.51)	
Alcohol use, % (SE)	77.31 (1.65)	81.65 (1.59)	76.99 (1.67)	76.12 (1.72)	74.31 (1.73)	**0.01**
Hypertension, % (SE)	52.89 (1.93)	41.00 (2.16)	53.02 (1.86)	56.56 (2.38)	61.45 (2.38)	** < 0.0001**
CVD, % (SE)	10.38 (1.18)	7.35 (1.04)	10.04 (1.10)	10.71 (1.32)	13.65 (1.35)	**0.002**
Stroke, % (SE)	2.88 (0.54)	2.18 (0.47)	2.24 (0.55)	2.52 (0.61)	4.69 (0.79)	**0.01**
CHD, % (SE)	4.21 (0.78)	2.82 (0.65)	4.22 (0.71)	4.41 (0.89)	5.46 (0.96)	**0.02**
CHF, % (SE)	2.85 (0.46)	1.40 (0.29)	2.96 (0.69)	3.06 (0.65)	4.04 (0.83)	**0.02**
ASCVD, % (SE)	9.44 (1.21)	6.87 (1.04)	9.04 (1.11)	9.96 (1.17)	12.08 (1.29)	**0.01**
Heart attack, % (SE)	4.50 (0.85)	3.44 (0.81)	9.32 (0.83)	4.34 (0.86)	4.75 (0.95)	0.39
Angina, % (SE)	3.36 (0.79)	1.87 (0.57)	4.69 (0.89)	3.20 (0.81)	3.74 (0.78)	0.06
DM, % (SE)	45.41 (2.05)	29.33 (1.80)	37.48 (2.12)	49.29 (2.07)	66.53 (2.31)	** < 0.0001**
PreDM, % (SE)	54.59 (2.13)	70.67 (1.80)	62.52 (2.12)	50.71 (2.07)	33.47 (2.31)	** < 0.0001**

LDL-C, Low-density lipoprotein cholesterol; HDL-C, High-density lipoprotein cholesterol; ACR, Urinary albumin: creatinine ratio; eGFR, estimated-glomerular filtration rate; BMI, Body mass index; PIR, Family income-poverty ratio; DM, Diabetes; PreDM, Prediabetes. CHF, Congestive heart failure; CVD, Cardiovascular disease; CHD, Congestive heart disease; ASCVD, Atherosclerotic cardiovascular disease

Bold value indicates the statistical significance

### Relationships of TyG index with the risk of CVD

Table 2 illustrates the relationship between the TyG index and the risk of CVD. Our findings revealed that a higher TyG index was linked to an elevated risk of CVD. This association was significant in both our unadjusted model (OR = 1.46, 95%CI 1.26–1.68, *p* < 0.0001) and the minimally adjusted model (OR = 1.48, 95%CI 1.26–1.77, *p* < 0.0001). Following full adjustment, a positive association between the TyG index and the CVD risk remained consistent (OR = 1.65, 95%CI 1.20–2.25, *p* = 0.002), signifying that each unit of the TyG index was associated with a 65% increase in CVD risk. When categorizing the TyG index into quartiles, in the fully adjusted models, participants in the highest TyG index showed a significant 63% increased risk of CVD compared to those in the lowest quartiles (OR = 1.63, 95%CI 1.03–2.56, *p* < 0.001).

**Table 2:** The association between TyG index and the risk of CVD

CVD	OR (95%CI)		
	Model 1	Model 2	Model 3
TyG index (continuous)	1.46 (1.26, 1.68), ***p* < 0.0001**	1.48 (1.26, 1.77), ***p* < 0.0001**	1.65 (1.20, 2.25) ***p* = 0.002**
TyG index (quartiles)			
Quartile 1	Reference	Reference	Reference
Quartile 2	1.51 (1.05, 2.17), ***p* = 0.03**	1.45 (1.01, 2.11), ***p* = 0.04**	1.37 (1.08, 1.66), ***p* = 0.03**
Quartile 3	1.64 (1.11, 2.16), ***p* = 0.03**	1.51 (1.07, 1.94), ***p* = 0.01**	1.49 (1.17, 1.80), ***p* = 0.03**
Quartile 4	1.99 (1.39, 2.86) ***p* < 0.001**	1.97 (1.34, 2.90), ***p* < 0.001**	1.63 (1.03, 2.56), ***p* < 0.001**

Model 1: No covariates were adjusted

Model 2: Age, gender, and race were adjusted

Model 3: Age, gender, race, education level, PIR, BMI, serum creatinine, serum uric acid, total cholesterol, HDL-C, LDL-C, ACR, eGFR, systolic blood pressure, diastolic blood pressure, hypertension, smoking and alcohol consumption status were adjusted

OR, odds ratio; 95%CI, 95% Confidence interval

Bold value indicates the statistical significance

No significant association between the TyG index and the risk of stroke, heart attack, and angina was found in this study (Tables 3, 4, and 5).

**Table 3:** The association between TyG index and the risk of Stroke

Stroke	OR (95%CI)		
	Model 1	Model 2	Model 3
TyG index (continuous)	1.56 (1.23, 1.97), ***p* < 0.001**	1.62 (1.26, 2.07), ***p* < 0.001**	1.54 (0.89, 2.67) *p* = 0.12
TyG index (quartiles)			
Quartile 1	Reference	Reference	Reference
Quartile 2	0.97 (0.55, 1.72), *p* = 0.91	0.98 (0.54, 1.75), *p* = 0.93	0.64 (0.33, 1.26), *p* = 0.20
Quartile 3	1.13 (0.58, 2.19), *p* = 0.71	1.09 (0.55, 2.18), *p* = 0.80	1.15 (0.73, 1.56), *p* = 0.41
Quartile 4	2.15 (1.24, 3.75) ***p* = 0.01**	2.23 (1.28, 3.88), ***p* = 0.005**	1.63 (0.75, 3.57), *p* = 0.22

Model 1: No covariates were adjusted

Model 2: Age, gender, and race were adjusted

Model 3: Age, gender, race, education level, PIR, BMI, serum creatinine, serum uric acid, total cholesterol, HDL-C, LDL-C, ACR, eGFR, systolic blood pressure, diastolic blood pressure, hypertension, smoking and alcohol consumption status were adjusted

OR, Odds ratio; 95%CI, 95% Confidence interval

Bold value indicates the statistical significance

**Table 4:** The association between TyG index and the risk of heart attack

Heart attack	OR (95%CI)		
	Model 1	Model 2	Model 3
TyG index (continuous)	1.20 (0.92, 1.56), *p* = 0.18	1.17 (0.86, 1.60), *p* = 0.30	1.24 (0.89, 1.74) *p* = 0.21
TyG index (quartiles)			
Quartile 1	Reference	Reference	Reference
Quartile 2	1.38 (0.94, 2.87), *p* = 0.08	1.51 (0.86, 2.64), *p* = 0.15	1.28 (0.72, 2.29), *p* = 0.40
Quartile 3	1.43 (0.67, 2.42), *p* = 0.46	1.16 (0.62, 2.20), *p* = 0.64	1.11 (0.59, 2.09), *p* = 0.59
Quartile 4	1.72 (0.76, 2.59)*p* = 0.28	1.31 (0.70, 2.46), *p* = 0.40	1.30 (0.66, 2.56), *p* = 0.44

Model 1: No covariates were adjusted

Model 2: Age, gender, and race were adjusted

Model 3: Age, gender, race, education level, PIR, BMI, serum creatinine, serum uric acid, total cholesterol, HDL-C, LDL-C, ACR, eGFR, systolic blood pressure, diastolic blood pressure, hypertension, smoking and alcohol consumption status were adjusted

OR, Odds ratio; 95%CI, 95% Confidence interval

**Table 5:** The association between TyG index and the risk of angina

Angina	OR (95%CI)		
	Model 1	Model 2	Model 3
TyG index (continuous)	1.30 (1.01, 1.67), ***p* = 0.04**	1.28 (0.95, 1.73), *p* = 0.11	1.03 (0.66, 1.61) *p* = 0.89
TyG index (quartiles)			
Quartile 1	Reference	Reference	Reference
Quartile 2	1.59 (1.31, 3.13), ***p* = 0.01**	1.42 (0.21, 2.85), *p* = 0.11	1.09 (0.51, 2.33), *p* = 0.82
Quartile 3	1.74 (1.23, 3.75), ***p* = 0.01**	1.52 (0.69, 3.35), *p* = 0.29	1.23 (0.52, 22.86), *p* = 0.64
Quartile 4	2.04 (1.35, 3.95) ***p* = 0.03**	1.89 (0.96, 3.74), *p* = 0.07	1.94 (0.91, 4.11), *p* = 0.08

Model 1: No covariates were adjusted

Model 2: Age, gender, and race were adjusted

Model 3: Age, gender, race, education level, PIR, BMI, serum creatinine, serum uric acid, total cholesterol, HDL-C, LDL-C, ACR, eGFR, systolic blood pressure, diastolic blood pressure, hypertension, smoking and alcohol consumption status were adjusted

OR, Odds ratio; 95%CI, 95% Confidence interval

Bold value indicates the statistical significance

For CHF, our study identified a positive association between the TyG index and an elevated likelihood of CHF with statistical significance (Table 6). In both our unadjusted model and minimally adjusted model, participants with higher TyG index levels exhibited an increased risk of CHF (Model 1: OR = 1.60, 95%CI 1.26–2.03, *p* < 0.001; Model 2: OR = 1.67, 95%CI 1.29–2.16, *p* < 0.001). After full adjustment, each unit increase in the TyG index was linked to a 47% increase in CHF risk (Model 3: OR = 1.47, 95%CI 1.03–2.09, *p* = 0.03). Even when considering the TyG index as quartiles, a statistically significant association persisted. Participants in the highest TyG index experienced a significant 107% higher risk compared to those in the lowest TyG index quartile (OR = 2.07, 95%CI 1.03–4.14, *p* = 0.04).

**Table 6:** The association between TyG index and the risk of CHF

CHF	OR (95%CI)		
	Model 1	Model 2	Model 3
TyG index (continuous)	1.60 (1.26, 2.03), ***p* < 0.001**	1.67 (1.29, 2.16), ***p* < 0.001**	1.47 (1.03, 2.09) ***p* = 0.03**
TyG index (quartiles)			
Quartile 1	Reference	Reference	Reference
Quartile 2	2.15 (1.17, 3.94), ***p* = 0.01**	2.10 (1.16, 3.81), ***p* = 0.02**	1.59 (1.11, 2.07), ***p* = 0.03**
Quartile 3	2.22 (1.23, 4.00), ***p* = 0.01**	2.18 (1.20, 3.94), ***p* = 0.01**	1.63 (1.08, 2.18), ***p* = 0.01**
Quartile 4	2.96 (1.65, 5.31) ***p* < 0.001**	3.03 (1.69, 5.45), ***p* < 0.001**	2.07 (1.03, 4.14), ***p* = 0.04**

Model 1: No covariates were adjusted

Model 2: Age, gender, and race were adjusted

Model 3: Age, gender, race, education level, PIR, BMI, serum creatinine, serum uric acid, total cholesterol, HDL-C, LDL-C, ACR, eGFR, systolic blood pressure, diastolic blood pressure, hypertension, smoking and alcohol consumption status were adjusted

OR, Odds ratio; 95%CI, 95% Confidence interval

Tables 7 and 8 revealed a significant risk increase between the TyG index and the risk of CHD (Model 3: OR 1.51, 95%CI 1.14–2.00, *p* = 0.005) and ASCVD (Model 3: OR 1.37, 95%CI 1.06–1.76, *p* = 0.02). In the sensitivity analyses, in fully adjusted Model 3, the highest quartile of the TyG index demonstrated an increase in the risk of both CHD (OR 1.70, 95%CI 1.12–3.19, *p* = 0.02) and ASCVD (OR 1.48, 95%CI 1.19–2.09, *p* = 0.01).

**Table 7:** The association between TyG index and the risk of CHD

CHD	OR (95%CI)		
	Model 1	Model 2	Model 3
TyG index (continuous)	1.46 (1.19, 1.81), ***p* < 0.001**	1.47 (1.14, 1.889), ***p* = 0.003**	1.51 (1.14, 2.00) ***p* = 0.005**
TyG index (quartiles)			
Quartile 1	Reference	Reference	Reference
Quartile 2	1.52 (1.09, 1.95), ***p* = 0.04**	1.31 (1.05, 1.57), ***p* = 0.03**	1.11 (1.02, 2.00), ***p* = 0.03**
Quartile 3	1.59 (1.11, 2.07), ***p* = 0.04**	1.38 (1.08, 1.68), ***p* = 0.03**	1.35 (1.09, 2.50), ***p* = 0.03**
Quartile 4	1.99 (1.13, 3.51) ***p* = 0.02**	1.77 (1.11, 3.17), ***P* = 0.04**	1.70 (1.12, 3.19), ***p* = 0.02**

Model 1: No covariates were adjusted

Model 2: Age, gender, and race were adjusted

Model 3: Age, gender, race, education level, PIR, BMI, serum creatinine, serum uric acid, total cholesterol, HDL-C, LDL-C, ACR, eGFR, systolic blood pressure, diastolic blood pressure, hypertension, smoking and alcohol consumption status were adjusted

OR, Odds ratio; 95%CI, 95% Confidence interval

Bold value indicates the statistical significance

**Table 8:** The association between TyG index and the risk of ASCVD

ASCVD	OR (95%CI)		
	Model 1	Model 2	Model 3
TyG index (continuous)	1.41 (1.20, 1.65), ***p* < 0.0001**	1.42 (1.18, 1.72), ***p* < 0.001**	1.37 (1.06, 1.76) ***p* = 0.02**
TyG index (quartiles)			
Quartile 1	Reference	Reference	Reference
Quartile 2	1.50 (1.05, 2.15), ***p* = 0.03**	1.42 (1.08, 2.06), ***p* = 0.04**	1.17 (1.06, 1.28), ***p* = 0.01**
Quartile 3	1.56 (1.09, 2.04), ***p* = 0.04**	1.63 (1.14, 2.18), ***p* = 0.04**	1.43 (1.15, 1.74), ***p* = 0.01**
Quartile 4	1.86 (1.25, 2.78) ***p* = 0.003**	1.81 (1.18, 2.76), ***p* = 0.01**	1.48 (1.19, 2.09), ***p* = 0.01**

Model 1: No covariates were adjusted

Model 2: Age, gender, and race were adjusted

Model 3: Age, gender, race, education level, PIR, BMI, serum creatinine, serum uric acid, total cholesterol, HDL-C, LDL-C, ACR, eGFR, systolic blood pressure, diastolic blood pressure, hypertension, smoking and alcohol consumption status were adjusted

OR, Odds ratio; 95%CI, 95% Confidence interval

Bold value indicates the statistical significance

### RCS analysis

**Fig. 2:**
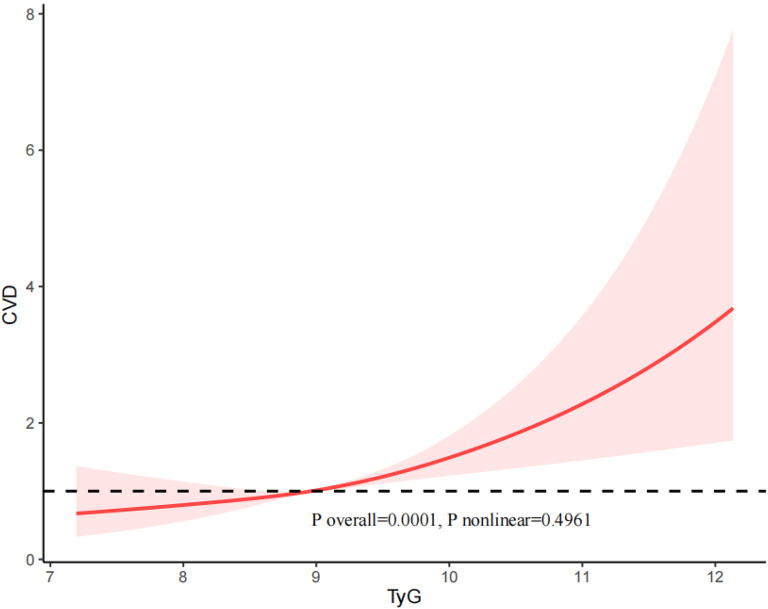
The restricted cubic spline (RCS) analysis between the TyG index and the risk of CVD

**Fig. 3:**
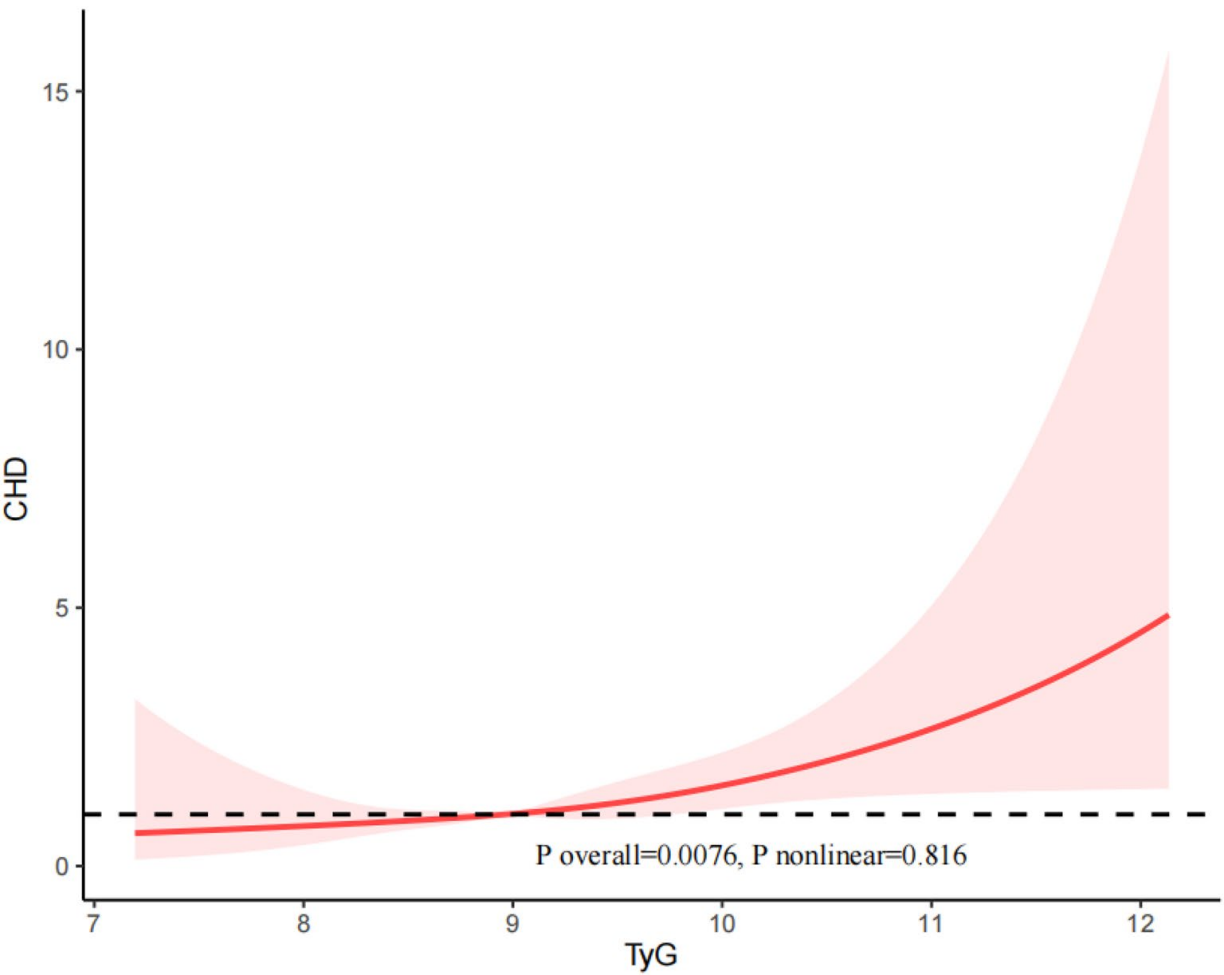
The restricted cubic spline (RCS) analysis between the TyG index and the risk of CHD

**Fig. 4:**
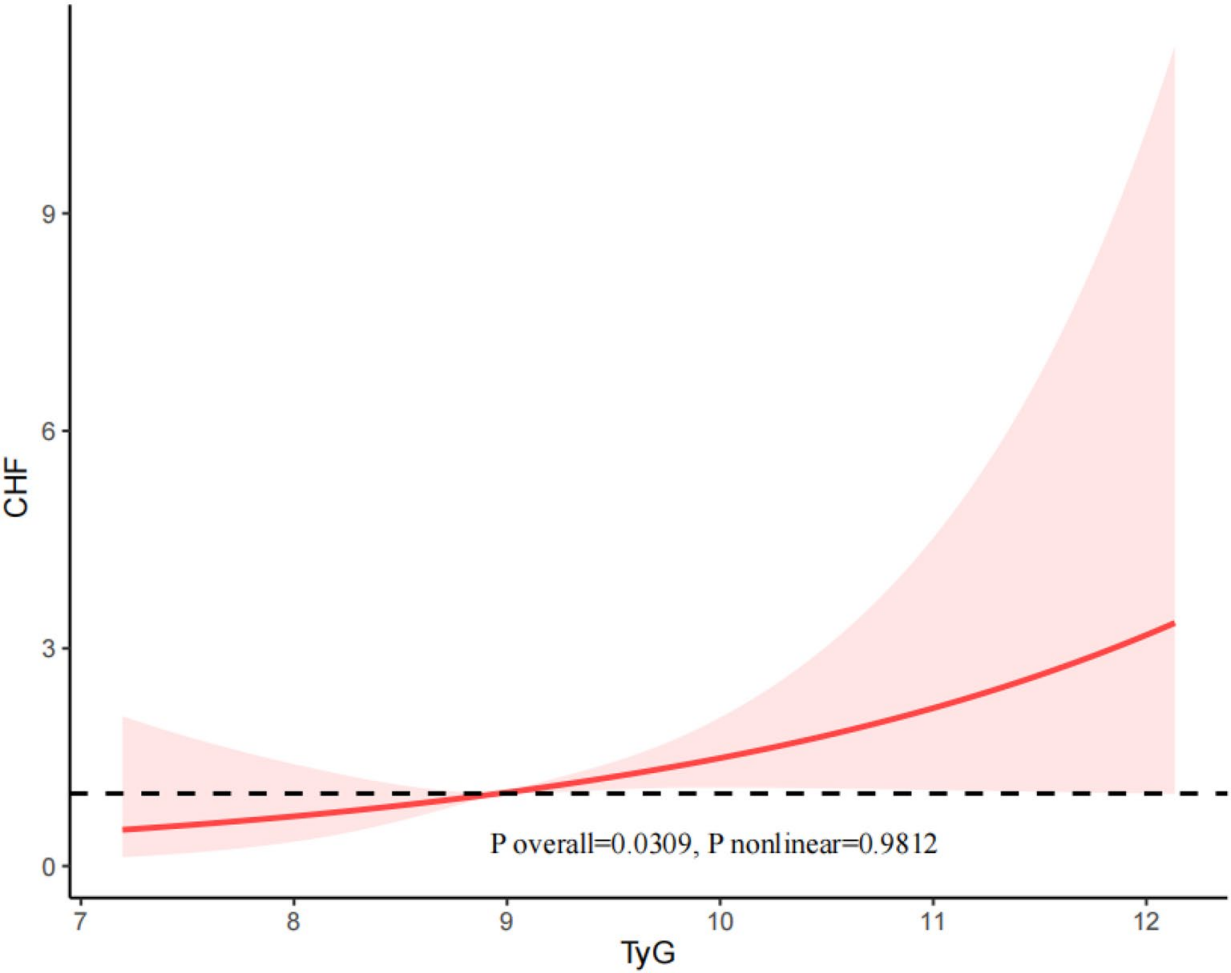
The restricted cubic spline (RCS) analysis between the TyG index and the risk of CHF

**Fig. 5:**
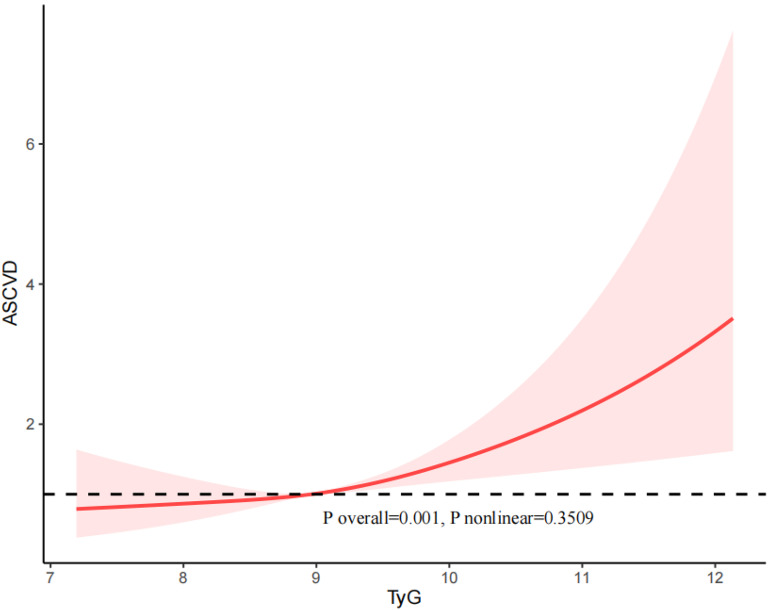
The restricted cubic spline (RCS) analysis between the TyG index and the risk of ASCVD

We utilized restricted cubic spline (RCS) curves to assess potential nonlinearity in the relationship between the TyG index and the risk of CVD, CHD, CHF, and ASCVD, as illustrated in Figs. 2, 3, 4, and 5. Our results indicated that there was an approximately linear relationship between the TyG index and the risk of CVD (P overall = 0.0001, P nonlinear = 0.4961), CHD (P overall = 0.0076, P nonlinear = 0.816), CHF (P overall = 0.0309, P nonlinear = 0.9812), and ASCVD (P overall = 0.001, P nonlinear = 0.3509).

Besides, in our study, we examined the population of individuals with diabetes and pre-diabetes separately. The results revealed a U-shaped relationship between the TyG index and both the risk of CVD (P nonlinear = 0.02583) and CHF (P nonlinear = 0.0208) in individuals with diabetes (Supplemental Figs. 1 and 3). The relationship between the TyG index and the risk of CHD (P nonlinear = 0.6958) and ASCVD (P nonlinear = 0.4331) was linear in patients with diabetes (Supplemental Figs. 2 and 4).

For the patients with pre-diabetes, the TyG index and the risk of CVD (P nonlinear = 0.6193), CHD (P nonlinear = 0.6768), CHF (P nonlinear = 0.1515), and ASCVD (P nonlinear = 0.9134) exhibited an approximately linear relationship.

### Subgroup analysis

**Fig. 6:**
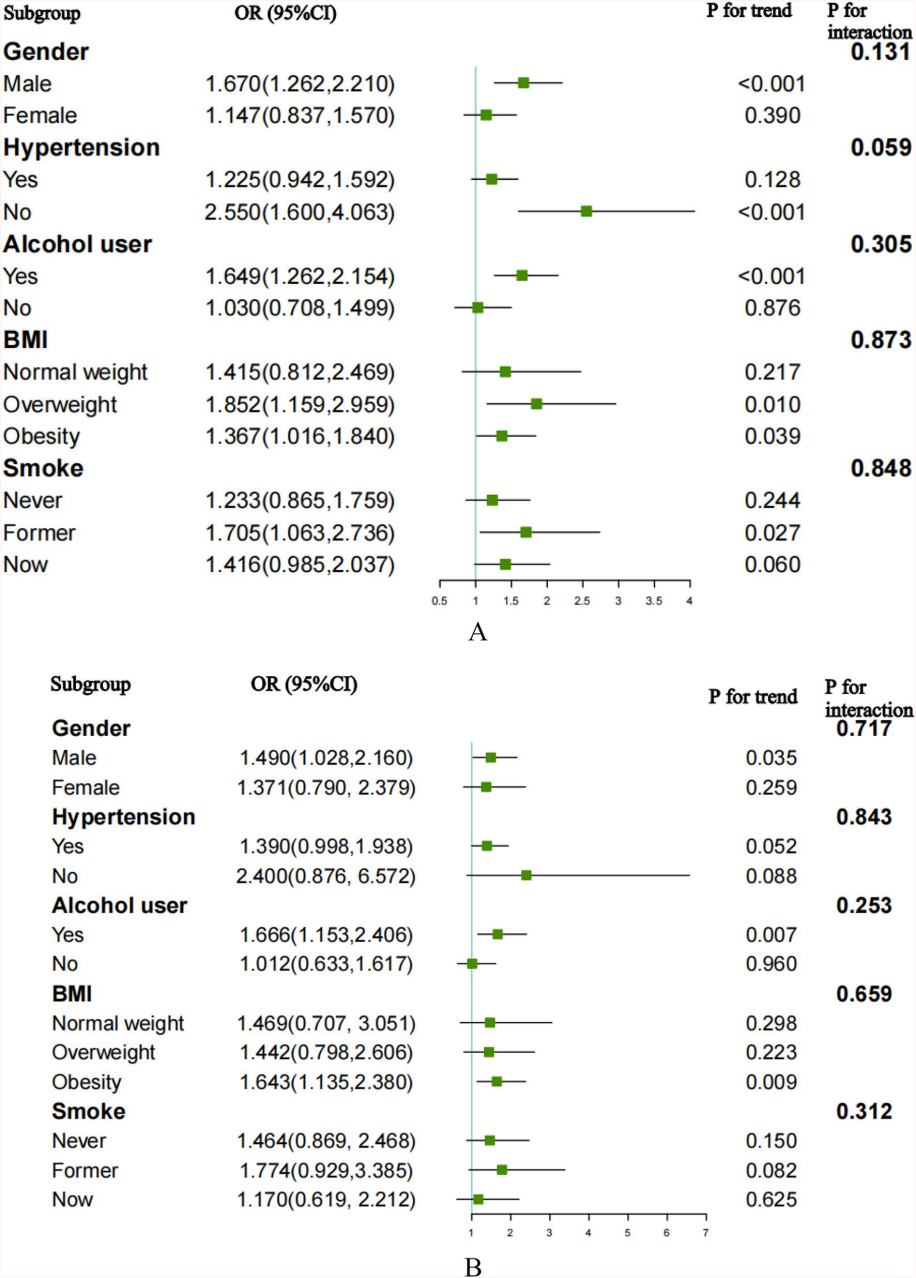

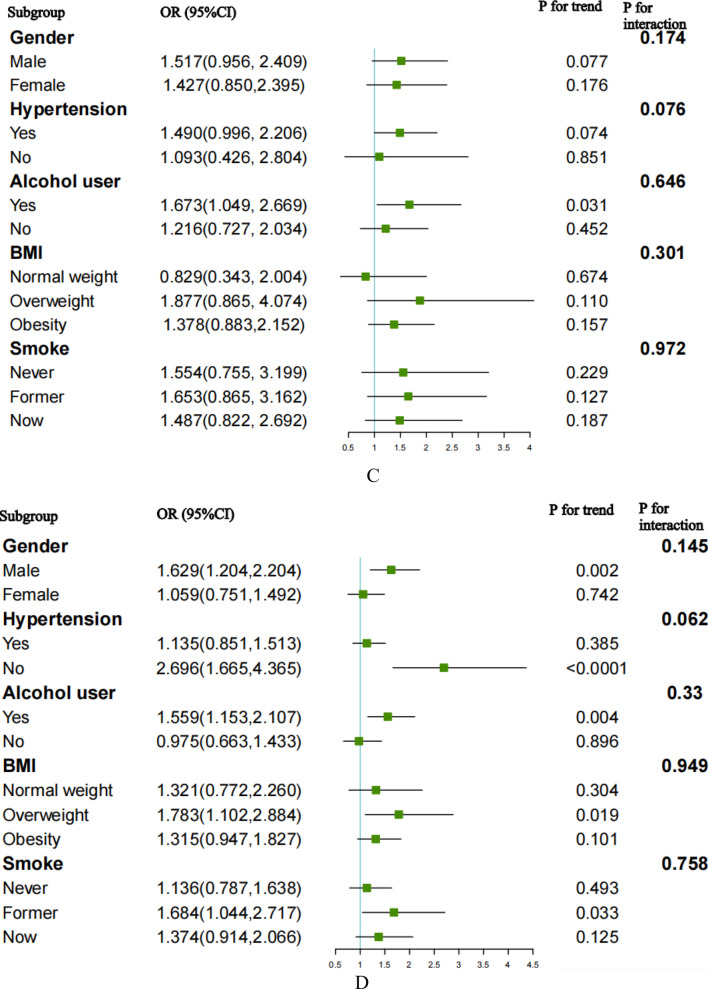
Subgroup analysis for the association between the TyG index and the risk of CVD, CHD, CHF, and ASCVD. **A** Subgroup analysis for the association between the TyG index and the risk of CVD. **B** Subgroup analysis for the association between the TyG index and the risk of CHD. **C** Subgroup analysis for the association between the TyG index and the risk of CHF. **D** Subgroup analysis for the association between the TyG index and the risk of ASCVD

In our subgroup analyses and interaction tests, we investigated the relationship between the TyG index and the risk of CVD, CHD, CHF, and ASCVD across different population subgroups (Fig. 6).

The risk of CVD increased in participants who were male (OR 1.670, 95%CI 1.262–2.210, *p* < 0.001), alcohol users (OR 1.649, 95%CI 1.262–2.154, *p* < 0.001), former smokers (OR 1.705, 95%CI 1.063–2.736, *p* = 0.027), overweight (OR 1.852, 95%CI 1.159–2.959, *p* = 0.010) and obese (OR 1.367, 95%CI 1.016–1.840, *p* = 0.039). Individuals without hypertension (OR 2.550, 95%CI 1.600–4.063, *p* < 0.001) also had an elevated risk of CVD.

For the risk of CHD, a positive relationship was observed in participants who were male (OR 1.490, 95%CI 1.028–2.160, *p* = 0.035) obese (OR 1.643, 95%CI 1.135–2.380, *p* = 0.009), and with a history of alcohol consumption (OR 1.666, 95%CI 1.153–2.406, *p* = 0.007). Alcohol users (OR 1.673, 95%CI 1.049–2.669, *p* = 0.031) were also reported to experience an increased risk of CHF.

Regarding ASCVD, the TyG index was associated with an increased risk of ASCVD, with significant correlations observed in males (OR 1.629, 95%CI 1.204–2.204, *p* = 0.002), those who were alcohol users (OR 1.559, 95%CI 1.153–2.107, *p* = 0.004), former smokers (OR 1.684, 95%CI 1.044–2.717, *p* = 0.033), overweight (OR 1.783, 95%CI 1.102–2.884, *p* = 0.019), and individuals without hypertension (OR 2.696, 95%CI 1.665–4.365, *p* < 0.0001).

Interaction tests did not reveal any significant influence of gender, BMI, alcohol use, smoking status, or hypertension on the association between the TyG index and the risk of CVD, CHD, CHF, and ASCVD (all *P* for interaction > 0.05). In conclusion, there was no significant interaction between the baseline TyG index and stratified variables.

### Relationships of TyG index with the risk of MetS

In our present study, the association between the TyG index and comorbid MetS in the U.S. population under 65 years of age with prediabetes or diabetes was further analyzed. For MetS, a positive correlation was observed between the TyG index and the likelihood of MetS with statistical significance (Supplemental Table 1). Both our crude (Model 1: OR = 6.84, 95%CI 5.63–8.30) and minimally adjusted models (Model 2: OR = 7.63, 95%CI 6.21–9.37) indicated that a higher TyG index was associated with an elevated likelihood of MetS. With full adjustment, a positive association between the TyG index and Mets remained stable (Model 3: OR = 7.22, 95%CI 5.75–9.06). Notably, even when stratifying the TyG index into quartiles, a significant association still persisted (Model 3: Quartile 2: OR = 1.45, 95%CI 1.14–1.83; Quartile 3: OR = 7.68, 95%CI 5.59–10.57; Quartile 4: OR = 26.54, 95%CI 18.57–37.92).

Stratified analysis was conducted across different gender, BMI, smoking, and drinking status groups to investigate potential heterogeneities. Supplemental Table 2 presents the associations between the TyG index and the likelihood of MetS within these groups. For MetS, a positive association was observed in both females (OR = 8.147, 95%CI 5.872–11.304) and males (OR = 7.708, 95%CI 5.560–10.685), both alcohol users (OR = 8.792, 95%CI 6.703–11.533) and non-alcohol users (OR = 4.521, 95%CI 3.111–6.571), among individuals with normal weight (OR = 25.781, 95%CI 11.241–59.128) and those classified as overweight (OR = 6.495, 95%CI 4.367–9.661) and obese (OR = 6.935, 95%CI 4.255–10.789), as well as those who were never smokers (OR = 7.440, 95%CI 5.556–9.963), former smokers (OR = 6.775, 95%CI 4.255–10.789) and current smokers (OR = 10.485, 95%CI 6.049–18.174). Additionally, the interaction test did not suggest significant differences among different stratifications, indicating that this positive association was not significantly influenced by gender, BMI, smoking, and drinking status (All *P* for interaction > 0.05) (Supplemental Table 2).

## Discussion

In this study, which included 4340 participants, we found that a higher TyG index was independently associated with an increased risk of CVD. In addition, we found a non-linear correlation between the TyG index and the risk of CVD and CHF in the diabetic population, as can be seen in the figure the threshold of the TyG index is 9.18, and the risk of CVD and CHF episodes increased significantly when the TyG index exceeded 9.18. There was no significant interaction between the baseline TyG index and stratified variables. In conclusion, our findings demonstrate that the TyG index can be used as a valid predictor of early cardiovascular risk in diabetic and prediabetic patients under 65 years of age in the United States. Besides, our study also revealed a positive association between the TyG index and comorbid MetS in the U.S. population under 65 years of age with prediabetes or diabetes. And results from the subgroup analysis suggested that this positive correlation was similar in different population settings.

Previous studies have evaluated the association between the TyG index and CVD risk in various populations. Liu et al. revealed that a heightened TyG index at baseline correlated with an increased risk of future CVD in postmenopausal women [27]. Similarly, Cai et al. identified a connection between a high TyG index and elevated risks of all-cause mortality and cardiovascular death among those at high risk of CVD [28]. Furthermore, Yu et al. established a positive association between a higher TyG index at baseline and the progression of carotid atherosclerosis, particularly in participants with a moderate stabilization trajectory [29]. Notably, TyG indices have also demonstrated positive associations with adverse cardiovascular outcomes in patients with stable cardiovascular disease [30]. Wan et al. reported findings supporting an association between elevated TyG indices and an increased risk of developing CVD and stroke [31]. Additionally, an elevated TyG index was found to be associated with a greater likelihood of CVD in non-malnourished populations [32]. These studies collectively underscore the significance of the TyG index as a valuable marker in assessing cardiovascular risk across various populations.

The influence of age on the correlation between the TyG index and CVD risk remains unclear, with most investigations focusing on middle-aged and older cohorts. Li et al. highlighted the utility of the TyG index in predicting CVD risk among individuals aged 60 and above [33]. Similarly, Hong et al. suggested its potential in early cardiovascular event detection for those aged 40 and older [34]. Research on Chinese middle-aged and elderly diabetic patients revealed an elevated TyG index associated with an increased CVD risk [35]. Moreover, a study of individuals over 65 exhibited a robust correlation between the TyG index and all-cause/CVD mortality [36]. Despite the prevailing focus on older populations, studies in younger patients have also indicated the predictive value of the TyG index for CVD risk. An Iranian population study demonstrated a significant association between the TyG index and increased CVD/coronary heart disease risk in younger individuals [37]. Another investigation noted a significant link between the TyG index and a higher incidence of heart failure in younger age groups [38]. In our current study, we observed a significant association between the TyG index and an elevated risk of early CVD in diabetic/pre-diabetic patients below 65 years of age. It is crucial to acknowledge that the impact of age on the association between the TyG index and CVD risk may vary, influenced by factors such as age-related comorbidities, biological aging, and the heterogeneity of patient populations. Furthermore, our observations revealed that none of the subgroups, encompassing variables such as sex, hypertension, BMI, smoking, and drinking status, exhibited significant changes in the relationship between TyG index and CVD incidence. This implies that our findings our findings are applicable to most individuals. These results contribute to a clearer understanding of the connection between baseline TyG levels and cardiovascular events. They underscore the TyG index's significance as a cost-effective and valuable early indicator, facilitating the identification of individuals who may be predisposed to CVD.

There appears to be a threshold effect in the association between the TyG index and CVD risk, indicating that both excessively high and low TyG levels may negatively impact health prognosis. One study has identified a negative correlation with the risk of CVD mortality when the TyG index falls below the threshold of 8.84, and a positive correlation with CVD mortality when the TyG index surpasses the threshold of 8.84 [39]. Notably, Zhao et al. demonstrated that patients with a TyG index in the intermediate range (8.72–9.15) exhibited the lowest risk of CVD mortality. In contrast, individuals in both the high and low TyG index groups experienced a significantly increased risk of CVD death [36]. The heightened risk of death in the high TyG index group may be attributed to hyperglycemia and hyperlipidemia, which are already major risk factors for cardiovascular events. On the other hand, the elevated risk of death in individuals with a low TyG index may result from hypoglycemia stimulating sympathetic nerves, leading to increased adrenaline levels and subsequent vasoconstriction, thereby elevating the risk of cardiovascular events [40]. In our study, while we did not observe a nonlinear relationship between the TyG index and CVD risk in the overall population, a U-shaped relationship between the TyG index and CVD risk was evident in the diabetic population.

The underlying mechanism of the predictive role of the TyG index for future CVD risk is unknown but may be related to the following factors. The prolonged hyperglycemic state resulting from decreased sensitivity to insulin in insulin resistance (IR) can initiate heightened glycosylation. This process contributes to collagen deposition and the formation of chronic fibrosis in myocardial tissues, resulting in the deterioration of cardiac function [7]. Concurrently, it induces oxidative stress, impairing the functionality of vascular endothelial cells and fostering the proliferation of vascular smooth muscle cells [41]. The heightened glycosylation triggered by IR also impacts the function of nitric oxide (NO), further exacerbating endothelial dysfunction [42–46]. Adipose tissue infiltration with proinflammatory mediators in adipocytes and macrophages fosters a local and systemic proinflammatory environment. This inflammatory state induces cardiac subcellular hypo-inflammation, leading to abnormalities in subcellular components such as oxidative stress, mitochondrial dysfunction, endoplasmic reticulum stress, and impaired calcium handling, ultimately resulting in impaired myocardial diastole [47–52]. Furthermore, IR promotes increased sympathetic excitability and heightened adrenaline secretion, creating a neural vicious cycle of humoral activation. This cycle leads to vasoconstriction, platelet aggregation, and, in severe cases, vascular stenosis [53–55]. The intricate interplay of these mechanisms underscores the multifaceted impact of IR on cardiovascular health.

We also evaluated the association between the TyG index and comorbid MetS in the U.S. population under 65 years of age with prediabetes or diabetes. MetS is composed of a spectrum of metabolic disorders including central obesity, hypertension, abnormal glucose metabolism, dyslipidemia, etc., which has become a global public health problem due to its increasing prevalence [56, 57]. IR assumes a pivotal role in the pathogenesis of MetS. Pathophysiological factors associated with IR manifest in metabolic disturbances among populations with MetS, including endothelial dysfunction, oxidative stress, and systemic metabolic inflammatory responses [58–61]. The TyG index serves as an early indicator for assessing IR and proves valuable in gauging the pro-inflammatory status of individuals. One study found that the TyG index was positively correlated with traditional cardiovascular risk factors such as FPG, TG, and TC, and negatively correlated with HDL-C, which are involved in the composition of the MetS [39]. A comprehensive longitudinal study in Korea underscored the TyG index's robust predictive capacity in assessing the long-term risk of MetS development [62]. The TyG index also demonstrated superior predictive capabilities for MetS compared to HOMA-IR [63]. Moreover, a meta-analysis of 13 observational studies corroborated the TyG index's optimal sensitivity and specificity for MetS screening, suggesting its potential as a valuable alternative biomarker for clinical follow-up management in MetS populations [64]. Notably, a significant elevation in the TyG index was observed in young adults diagnosed with MetS [65]. Therefore, TyG index assessment will contribute to more convenient and effective screening of high-risk individuals in patients with MetS.

The strength of our research is based on the NHANES database. The expansive size of the sample ensures robust statistical power, while its representation of the entire U.S. population assures a heightened level of external validity. Additionally, all variables are meticulously gathered in a standardized and homogeneous manner. We mitigated confounding bias through the meticulous adjustment of covariates, thereby augmenting the robustness of our findings. However, it is imperative to acknowledge certain limitations inherent in our study. Firstly, it is imperative to note that our research is confined to a single-center observational study, precluding the establishment of causality. Additionally, the study cohort predominantly comprises individuals from the United States, thereby constraining its generalizability on a global scale. Secondly, despite our efforts to account for potential confounding covariates, the possibility of residual confounders cannot be completely dismissed. Moreover, the TyG index was derived from a solitary baseline blood sample, leaving the impact of variations in the TyG index throughout the follow-up period on the risk of cardiovascular events uncertain.

## Conclusion

The findings of our study indicate that the TyG index could serve as a potentially valuable predictor of CVD risk in individuals under 65 years of age with diabetes or prediabetes in the United States. Furthermore, our results revealed a nonlinear relationship between the TyG index and the risk of both CVD and CHF in patients with diabetes. This has important clinical implications for the early identification of CVD risk in non-elderly individuals with diabetes and prediabetes. In addition, our study also revealed a positive association between the TyG index and comorbid MetS in the U.S. population under 65 years of age with prediabetes or diabetes. The study's insights suggest that future research should be directed towards exploring whether interventions targeting the TyG index can lead to improvements in clinical prognosis for younger patients with diabetes and prediabetes.

### Supplementary Information

Supplementary Material 1.

Supplementary Material 2.

Supplementary Material 3.

Supplementary Material 4.

Supplementary Material 5.

Supplementary Material 6.

## Data Availability

Publicly available datasets were analyzed in this study. This data can be found here: https://www.cdc.gov/nchs/nhanes/index.htm.
