# Supplementary material for: The association between the triglyceride–glucose index and the risk of cardiovascular disease in US population aged ≤ 65 years with prediabetes or diabetes: a population-based study
Source: Cardiovasc Diabetol. 2024 May 13;23:168. doi: 10.1186/s12933-024-02261-8 (PMC11092030; doi:10.1186/s12933-024-02261-8)

Supplemental Figure 4 The restricted cubic spline (RCS) analysis between the TyG index and the risk of ASCVD in participants with diabetes (DM) or prediabetes (PreDM).

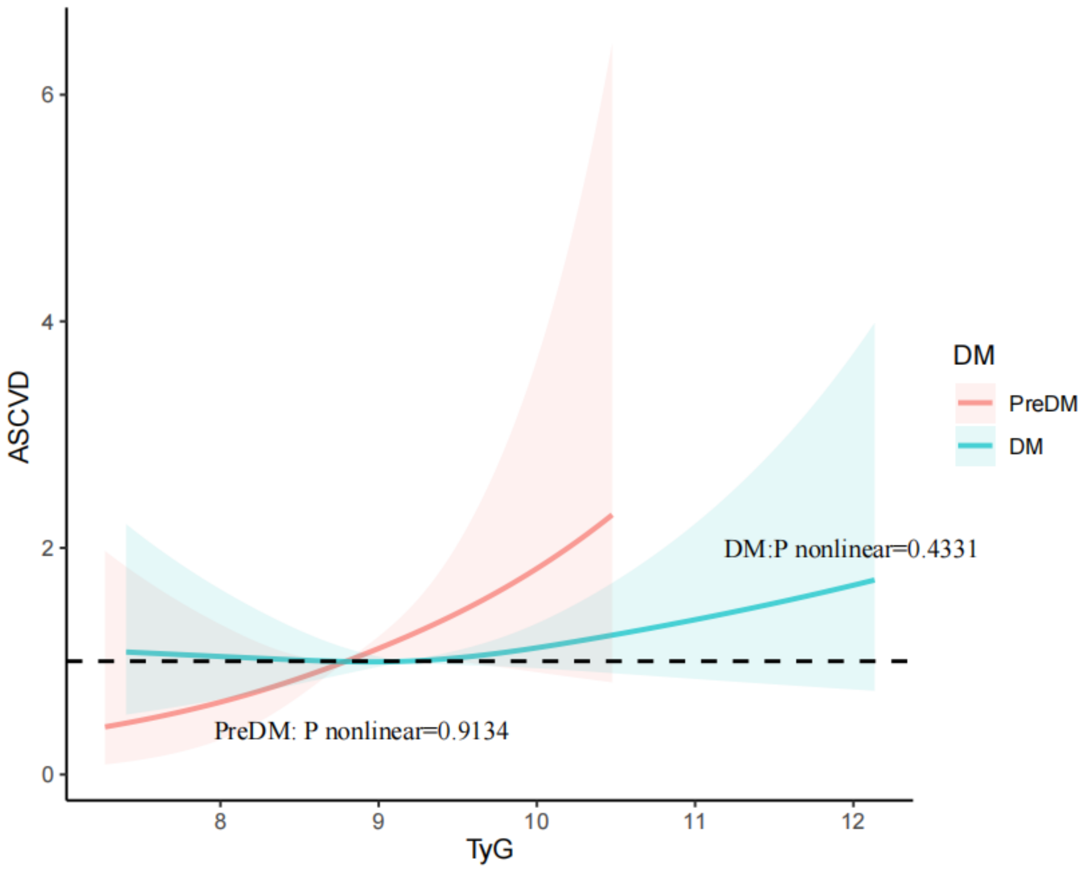

Supplement: Supplementary file 4 — Supplementary Material 4. [file 12933_2024_2261_MOESM4_ESM.pdf]
