# Supplementary material for: The association between the triglyceride–glucose index and the risk of cardiovascular disease in US population aged ≤ 65 years with prediabetes or diabetes: a population-based study
Source: Cardiovasc Diabetol. 2024 May 13;23:168. doi: 10.1186/s12933-024-02261-8 (PMC11092030; doi:10.1186/s12933-024-02261-8)
Supplement: Supplementary file 5 — Supplementary Material 5. [file 12933_2024_2261_MOESM5_ESM.docx]

| MetS | OR (95%CI) |  |  |
| --- | --- | --- | --- |
|  | Model 1 | Model 2 | Model 3 |
| TyG index (continuous) | 6.84 (5.63, 8.30), **p<0.0001** | 7.63 (6.21, 9.37),  **p<0.0001** | 7.22 (5.75, 9.06)  **p<0.0001** |
| TyG index (quartiles) |  |  |  |
| Quartile 1 | Reference | Reference | Reference |
| Quartile 2 | 1.93 (1.55, 2.39),  **p<0.0001** | 2.08 (1.67, 2.58),  **p<0.0001** | 1.45 (1.14, 1.83), **p=0.002** |
| Quartile 3 | 7.89 (6.01, 10.35),  **p<0.0001** | 8.67 (6.40, 11.73),  **p<0.0001** | 7.68 (5.59, 10.57), **p<0.0001** |
| Quartile 4 | 21.49 (15.61, 29.58)  **p<0.0001** | 27.24 (19.87, 37.34),  **p<0.0001** | 26.54 (18.57, 37.92),  **p<0.0001** |

**Supplemental Table 1 The association between TyG index and the risk of Mets.**

Mets: metabolic syndrome; OR: odds ratio; 95%CI: 95% confidence interval

Model 1: No covariates were adjusted.

Model 2: Age, gender, and race were adjusted.

Model 3: Age, gender, race, education level, PIR, BMI, serum creatinine, serum uric acid, ACR, eGFR, smoking and alcohol consumption status were adjusted.
