# Supplementary material for: The association between the triglyceride–glucose index and the risk of cardiovascular disease in US population aged ≤ 65 years with prediabetes or diabetes: a population-based study
Source: Cardiovasc Diabetol. 2024 May 13;23:168. doi: 10.1186/s12933-024-02261-8 (PMC11092030; doi:10.1186/s12933-024-02261-8)
Supplement: Supplementary file 6 — Supplementary Material 6. [file 12933_2024_2261_MOESM6_ESM.docx]

| MetS | OR (95% CI) | P for trend | P for interaction |
| --- | --- | --- | --- |
| Gender |  |  | 0.382 |
| Female | 8.147 (5.872, 11.304) | **<0.0001** |  |
| Male | 7.708 (5.560, 10.685) | **<0.0001** |  |
| BMI |  |  | 0.098 |
| Normal weight | 25.781 (11.241, 59.128) | **<0.0001** |  |
| Overweight | 6.495 (4.367, 9.661) | **<0.0001** |  |
| Obesity | 6.935 (5.255, 9.152) | **<0.0001** |  |
| Smoke |  |  | 0.738 |
| Never | 7.440 (5.556, 9.963) | **<0.0001** |  |
| Former | 6.775 (4.255, 10.789) | **<0.0001** |  |
| Now | 10.485 (6.049, 18.174) | **<0.0001** |  |
| Alcohol user |  |  | 0.054 |
| Yes | 8.792 (6.703, 11.533) | **<0.0001** |  |
| No | 4.521 (3.111, 6.571) | **<0.0001** |  |

**Supplemental Table 2 Subgroup analysis for the association between the TyG index and the risk of MetS**

Mets: metabolic syndrome; OR: odds ratio; 95%CI: 95% confidence interval
